# Characterization and Sequencing of an H6N6 Avian Influenza Virus Isolated from Sansui Sheldrake Ducks in Guizhou, Southwestern China

**DOI:** 10.1128/genomeA.00351-16

**Published:** 2016-05-12

**Authors:** Zhiqiang Duan, Jiaqi Chen, Xinqin Ji, Houqiang Xu, Yong Ruan, Jiafu Zhao

**Affiliations:** a Key Laboratory of Animal Genetics, Breeding and Reproduction in The Plateau Mountainous Region, Ministry of Education, Guizhou University, Guiyang, China; b College of Animal Science, Guizhou University, Guiyang, China

## Abstract

Here, we report the complete genome sequence of an H6N6 avian influenza virus (AIV) isolated from Sansui Sheldrake ducks in Guizhou Province, China, in 2014. Phylogenetic analysis showed that the H6N6 virus was a reassortant virus derived from three different H6 subtype lineages. The finding of this study will help us understand the epidemiology and the evolutionary characteristics of H6 subtypes of AIV in ducks in southwestern China.

## GENOME ANNOUNCEMENT

Waterfowls are known as a giant genetic reservoir for avian influenza virus (AIV), and to date, all 16 hemagglutinin (HA) and 9 neuraminidase (NA) subtypes of AIV have been isolated from waterfowl species (1). The H6 subtypes of AIV are the most abundantly detected influenza subtypes in wild and domestic aquatic avian species, and they have a much broader host range than any other AIV subtypes (2, 3). Recent studies concerning human H5N6 infection have demonstrated that the isolated H5N6 viruses are the avian-originated viruses and belong to novel reassortants between H5N1 and H6N6 viruses (4, 5). Therefore, it is important to enhance the epidemiological surveillance of the H6N6 subtypes of AIV in ducks to understand the emergence of novel reassortants with potential threat to human health.

In this study, an H6N6 subtype AIV strain, A/duck/Guizhou/013/2014 (H6N6), was isolated from apparently healthy Sansui Sheldrake ducks in Guizhou Province, China, in 2014. All eight gene segments of the H6N6 AIV were sequenced by DNA direct sequencing. Editing and analysis of the sequence data were performed with Lasergene 7.1 and MEGA 6.0.

The full lengths of the polymerase basic 2 (PB2), PB1, polymerase acidic (PA), HA, nucleoprotein (NP), NA, matrix (M), and nonstructural (NS) genes were 2,341, 2,341, 2,233, 1,744, 1,565, 1,465, 1,027, and 890 nucleotides, respectively. Phylogenetic analysis showed that the nucleotide sequence identities of the HA and NA genes with that of the H6N6 isolates A/duck/Jiangsu/022/2009 (H6N6) and A/duck/Fujian/8089/2007 (H6N6) were 97% and 98%, respectively. The PB2 and PB1 genes were most closely similar to the foreign isolates A/duck/Yamagata/06/2014 (H6N6) and A/duck/Vietnam/LBM6/2011 (H6N6), with which they share 97% and 98% nucleotide homology, respectively. The other four genes were found to be more similar to those of southern China H6N6 strains (up to 97% to 99% nucleotide homology). In addition, the phylogenetic trees revealed that all eight gene segments of this isolate belonged to the Eurasian avian lineage, but the HA and PB2 genes belonged to the sequence type 2853 (ST2853)-like and HN573-like lineages, respectively, whereas the other genes belonged to ST339-like lineage. These results indicate that the H6N6 virus was a reassortant virus resulting from three different H6 subtype lineages.

Amino acid sequence analysis showed that the cleavage site motif between HA1 and HA2 was PQIETR↓G; this is a typical characteristic of low-pathogenicity AIV (6). Similar to H5 HA, the receptor binding sites in the HA protein were Q224 and G226 (H6 numbering), which would preferentially bind to alpha-linked (2, 3) sialic acid receptor (7, 8). Analysis of the NA sequences revealed that there was no mutation at the H275Y position, indicating that the virus may be sensitive to neuraminidase inhibitor drugs (9). The PB2 amino acid residues at positions 627 and 701 still remained the characteristics of AIV (E627 and D701), so it could not replicate in mammalian hosts (10, 11). In addition, there were no mutations at positions L26, I27, A30, S31, and G34 in the M2 protein, suggesting that this strain is not amantadine resistant (12). These findings will help us understand the epidemiology and the evolutionary characteristics of H6 subtype AIVs circulating in ducks in southwestern China.

### Nucleotide sequence accession numbers.

The complete genome sequences of A/duck/Guizhou/013/2014 (H6N6) have been deposited in GenBank under the accession numbers KU762356 to KU762363.

## References

[1] Kaleta EF, Hergarten G, Yilmaz A 2005 Avian influenza A viruses in birds—an ecological, ornithological and virological view. Dtsch Tierarztl Wochenschr 112:448–456. 16425630

[2] Spackman E, Stallknecht DE, Slemons RD, Winker K, Suarez DL, Scott M, Swayne DE 2005 Phylogenetic analyses of type A influenza genes in natural reservoir species in North America reveals genetic variation. Virus Res 114:89–100. doi:10.1016/j.virusres.2005.05.013. 16039745

[3] Peng Y, Xie ZX, Liu JB, Pang YS, Deng XW, Xie ZQ, Xie LJ, Fan Q, Luo SS 2013 Epidemiological surveillance of low pathogenic avian influenza virus (LPAIV) from poultry in Guangxi Province, southern China. PLoS One 8:e77132. doi:10.1371/journal.pone.0077132. 24204754 PMC3813733

[4] Mok CKP, Da Guan W, Liu XQ, Lamers MM, Li XB, Wang M, Zhang TJS, Zhang QL, Li ZT, Huang JC, Lin JY, Zhang YH, Zhao P, Lee HHY, Chen L, Li YM, Peiris JSM, Chen RC, Zhong NS, Yang ZF 2015 Genetic characterization of highly pathogenic avian influenza A(H5N6) virus, Guangdong, China. Emerg Infect Dis 21:2268–2271. doi:10.3201/eid2112.150809. 26584075 PMC4672456

[5] Bi Y, Mei K, Shi W, Liu D, Yu X, Gao Z, Zhao L, Gao GF, Chen J, Chen Q 2015 Two novel reassortants of avian influenza A (H5N6) virus in China. J Gen Virol 96:975–981. doi:10.1099/vir.0.000056. 25604926

[6] Li M, Xie ZX, Xie ZQ, Luo SS, Xie LJ, Huang L, Deng XW, Huang JL, Zhang YF, Zeng TT, Wang S 2015 Molecular characteristics of H6N6 influenza virus isolated from pigeons in Guangxi, southern China. Genome Announc 3(6):e01422-15. doi:10.1128/genomeA.01422-15. 26634763 PMC4669404

[7] Matrosovich M, Tuzikov A, Bovin N, Gambaryan A, Klimov A, Castrucci MR, Donatelli I, Kawaoka Y 2000 Early alterations of the receptor-binding properties of H1, H2 and H3 avian influenza virus hemagglutinins after their introduction into mammals. J Virol 74:8502–8512. 10954551 10.1128/jvi.74.18.8502-8512.2000 PMC116362

[8] Wu H, Wu N, Peng X, Jin C, Lu X, Cheng L, Yao H, Li L 2014 Molecular characterization and phylogenetic analysis of H3 subtype avian influenza viruses isolated from domestic ducks in Zhejiang Province in China. Virus Genes 49:80–88. doi:10.1007/s11262-014-1065-9. 24748106

[9] Suzuki H, Saito R, Masuda H, Oshitani H, Sato M, Sato I 2003 Emergence of amantadine-resistant influenza A viruses: epidemiological study. J Infect Chemother 9:195–200. doi:10.1007/s10156-003-0262-6. 14513385

[10] Massin P, van der Werf S, Naffakh N 2001 Residue 627 of PB2 is a determinant of cold sensitivity in RNA replication of avian influenza viruses. J Virol 75:5398–5404. doi:10.1128/JVI.75.11.5398-5404.2001. 11333924 PMC114948

[11] Li Z, Chen H, Jiao P, Deng G, Tian G, Li Y, Hoffmann E, Webster RG, Matsuoka Y, Yu K 2005 Molecular basis of replication of duck H5N1 influenza viruses in a mammalian mouse model. J Virol 79:12058–12064. doi:10.1128/JVI.79.18.12058-12064.2005. 16140781 PMC1212590

[12] Furuse Y, Suzuki A, Oshitani H 2009 Large-scale sequence analysis of M gene of influenza A viruses from different species: mechanisms for emergence and spread of amantadine resistance. Antimicrob Agents Chemother 53:4457–4463. doi:10.1128/AAC.00650-09. 19651904 PMC2764202

